# Comparative study on high-pressure physical properties of monoclinic MgCO_3_ and Mg_2_CO_4_

**DOI:** 10.1038/s41598-022-24033-8

**Published:** 2022-11-14

**Authors:** Zi-Jiang Liu, Tian Li, Xiao-Wei Sun, Cai-Rong Zhang, Jia-Qi Ju

**Affiliations:** 1 School of Mathematics and Physics, Lanzhou Jiaotong University, Lanzhou, 730070 China; 2 Department of Applied Physics, Lanzhou University of Technology, Lanzhou, 730050 China

**Keywords:** Solid Earth sciences, Geophysics, Mineralogy

## Abstract

The physical properties of Mg-carbonate at high temperature and pressure are crucial for understanding the deep carbon cycle. Here, we use first-principles calculations to study the physical properties of MgCO_3_-*C*2/*m* and Mg_2_CO_4_-*P*2_1_/*c* under high pressure. The research shows that the structure and equation of state of MgCO_3_-*C*2/*m* are in good agreement with the experimental results, and the phase transition pressure of Mg_2_CO_4_ from *pnma* to *P*2_1_/*c* structure is 44.66 GPa. By comparing the elastic properties, seismic properties and anisotropy of MgCO_3_-*C*2/*m* and Mg_2_CO_4_-*P*2_1_/*c*, it is found that the elastic modulus and sound velocity of Mg_2_CO_4_-*P*2_1_/*c* are smaller than those of MgCO_3_-*C*2/*m*, while the anisotropy is larger than that of MgCO_3_-*C*2/*m*. These results indicate that Mg_2_CO_4_-*P*2_1_/*c* exists in the deep mantle and may be the main reason why carbonate cannot be detected. The minimum thermal conductivity of MgCO_3_-*C*2/*m* and Mg_2_CO_4_-*P*2_1_/*c* is the largest in the [010] direction and the smallest in the [001] direction. The thermodynamic properties of MgCO_3_-*C*2/*m* and Mg_2_CO_4_-*P*2_1_/*c* are predicted using the quasi-harmonic approximation (QHA) method.

## Introduction

Magnesite (space group $R\overline{3} c$) is subducted into the deep mantle as the primary carbon carrier, and its high-temperature and high-pressure physical properties are critical for understanding the deep carbon cycle^1,2^. Previous studies have mainly focused on the structural phase transition of MgCO_3_-$R\overline{3} c$ under high-temperature and high-pressure conditions, transforming to monoclinic MgCO_3_ (space group *C*2/*m*) at around 80 GPa. Oganov et al. used the USPEX method to predict for the first time that MgCO_3_-*C*2/*m* is most stable in the lower mantle greater than 82.4 GPa^3^. MgCO_3_-*C*2/*m* has 30 atoms, in which adjacent oxygen atoms form tetrahedra around carbon atoms, and Mg atoms are in octet and tenfold coordination. Subsequently, the structure of MgCO_3_-*C*2/*m* was verified experimentally^4–6^ and theoretically^7–13^. Recently, Gavryushkin et al. used USPEX and AIRSS methods to find that MgCO_3_ reacts with MgO to form Mg_2_CO_4_, which has two structures, orthorhombic (space group *Pnma*) and monoclinic (space group *P*2_1_/*c*), and its structure is *P*2_1_/*c* when the pressure is higher than 50 GPa^14^. Their experiments later confirmed the existence of Mg_2_CO_4_-*P*2_1_/*c* at the temperature and pressure of the Earth's lower mantle^15^. Mg_2_CO_4_-*P*2_1_/*c* has 28 atoms, it is isostructural to *β*-Ca_2_SiO_4_^16^, and the atoms at the two positions Mg(1) and Mg(2) are six-coordinated, with octahedral coordination polyhedra. Earlier reports^17–20^, although the structure of Mg_2_CO_4_ was not determined, believed that it is stable at high pressure.

To understand the carbon cycle in the deep earth, it is crucial to study the structure, phase transition, equations of state, elasticity, thermodynamics, and thermal conductivity of MgCO_3_-*C*2/*m* and Mg_2_CO_4_-*P*2_1_/*c* under high pressure. Recently, Maeda et al. measured the equation of state of MgCO_3_-*C*2/*m* at high pressure^6^. Since it is extremely difficult to measure the elastic constants, thermodynamic parameters, and thermal conductivity of minerals experimentally, the properties of MgCO_3_-*C*2/*m* and Mg_2_CO_4_-*P*2_1_/*c* have not been reported experimentally. Even the elastic constant of MgCO_3_-$R\overline{3} c$ can only be measured to 13.7 GPa^21^, and its thermodynamic properties are only at low pressure, and the results at high pressure are extrapolated^22–24^.

In the present work, the structures, phase transitions, equations of state, elastic properties, seismic properties, and minimum thermal conductivity of MgCO_3_-*C*2/*m* and Mg_2_CO_4_-*P*2_1_/*c* at high pressure are investigated using first-principles calculations and compared with the available experimental and theoretical results. The QHA method is adopted to research the thermodynamic properties of MgCO_3_-*C*2/*m* and Mg_2_CO_4_-*P*2_1_/*c*.

## Methods

First-principles calculations are used to investigate the high-pressure physical properties of MgCO_3_-*C*2/*m* and Mg_2_CO_4_-*P*2_1_/*c* using projector-augmented wave (PAW)^25^ as implemented in VASP^26,27^. The electronic configurations: 2p^6^3s^2^ for Mg, 2s^2^2p^2^ for C and 2s^2^2p^4^ for O are considered for the valence electrons. The Perdew–Burke–Ernzerhof modified solid (PBEsol) in the generalized gradient approximation (GGA)^28^ describes the exchange and correlation potentials. The generation of k-point mesh and the calculation of elastic properties utilize the vaspkit program^29^. The cutoff energy for the plane wave is set to 900 eV. The *k*-points mesh of MgCO_3_-*C*2/*m* and Mg_2_CO_4_-*P*2_1_/*c* are set to 4 × 5 × 5 and 13 × 9 × 7 using the Monkhorst–Pack scheme^30^, respectively. The thermodynamic properties are calculated by the QHA method^31^.

## Results and discussion

### Structural parameters, phase transition and equation of state

**Figure 1:**
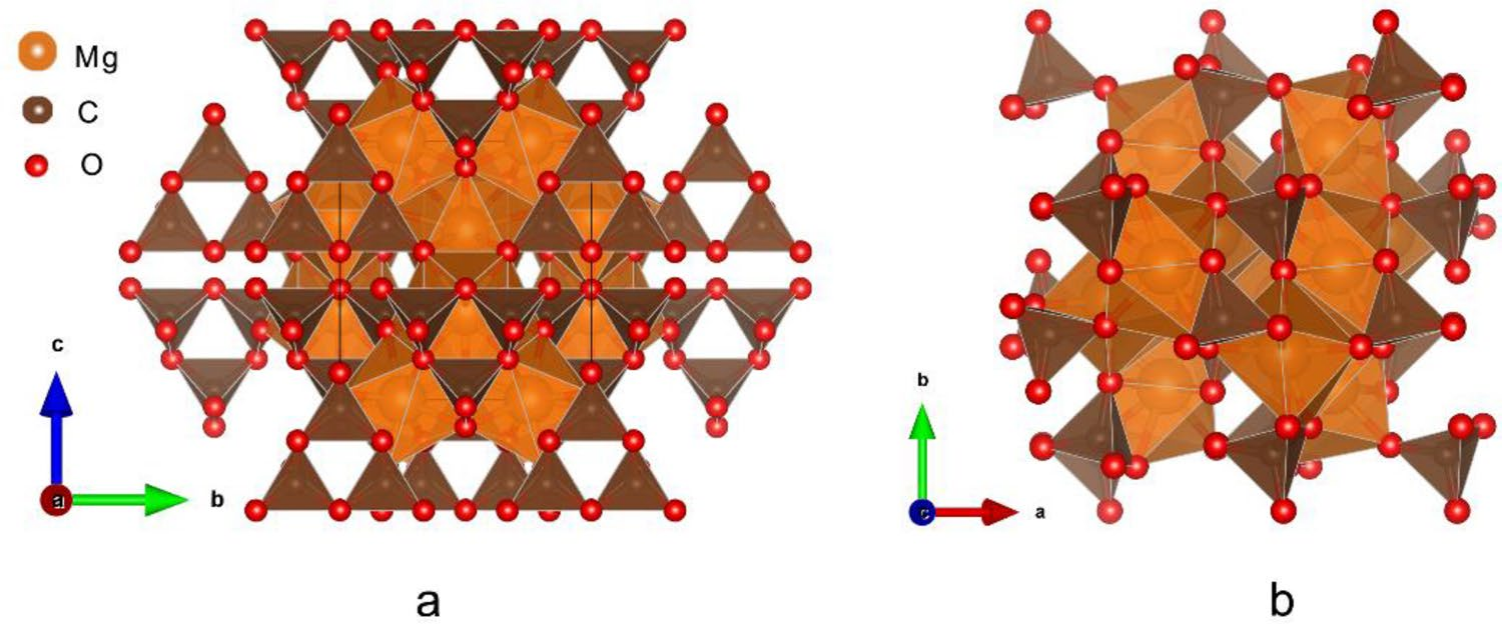
Crystal structures of MgCO_3_-*C*2/*m* (**a**) and Mg_2_CO_4_-*P*2_1_/*c* (**b**) in unit cell. The crystal structures are drawn by VESTA^32^.

The crystal structures of MgCO_3_-*C*2/*m* and Mg_2_CO_4_-*P*2_1_/*c* in the unit cell are shown in Fig. 1. The optimized lattice parameters are summarized in Table 1 and compared with available experimental and previously calculated results. At 110 GPa, the calculated results of MgCO_3_-*C*2/*m* are consistent with the experimental results^5^. The results for Mg_2_CO_4_-*P*2_1_/*c* at 100 GPa agree well with the previous calculations^14^.

**Table 1:** Lattice parameters of MgCO_3_-*C*2/*m* and Mg_2_CO_4_-*P*2_1_/*c* at 110 GPa and 100 GPa, respectively, compared with experimental and previous calculations.

Method	a (Å)	b (Å)	c (Å)	*β*	V(Å^3^)
**MgCO**_**3**_**-*C*2/*m***					
This work	8.104	6.493	6.884	103.893	351.61
Binck et al.^5^	8.117	6.510	6.911	103.858	354.64
**Mg**_**2**_**CO**_**4**_**-*P*2**_**1**_**/*c***					
This work	4.383	5.358	8.293	117.56	172.65
Gavryushkin et al.^14^	4.408	5.383	8.345	117.65	175.39

**Figure 2:**
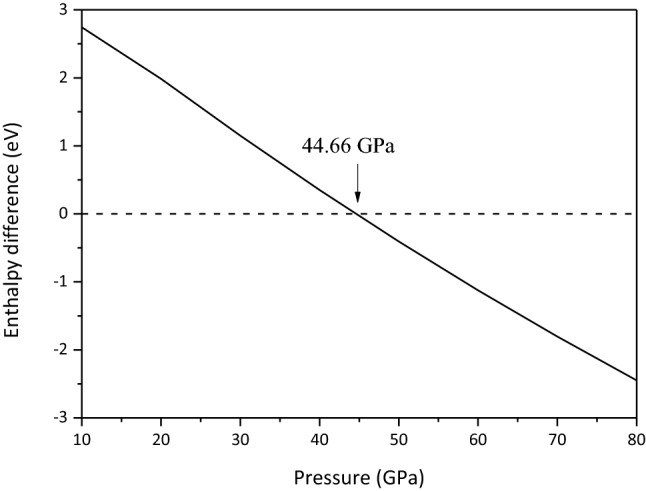
Enthalpy difference between Mg_2_CO_4_-*P*2_1_/*c* and Mg_2_CO_4_-*Pnma*.

As shown in Fig. 2, the present calculated transition pressure from Mg_2_CO_4_-*Pnma* to Mg_2_CO_4_-*P*2_1_/*c* is 44.66 GPa, while Gavryushkin et al. calculated 52 GPa^14^. This error may be caused by the use of different exchange correction functions, PBEsol is used in the present work, while PBE was used by Gavryushkin et al.^14^. The accuracy of using PBEsol to calculate the properties of Mg-carbonate has been examined in the previous studies^13^. In the previous study, MgCO_3_-*C*2/*m* was stable in the lower mantle above 80 GPa^3,5–13,33^. Therefore, in order to facilitate comparison, the high-pressure properties of MgCO_3_-*C*2/*m* and Mg_2_CO_4_-*P*2_1_/*c* in the pressure range of 50–140 GPa are investigated in this work.

**Figure 3:**
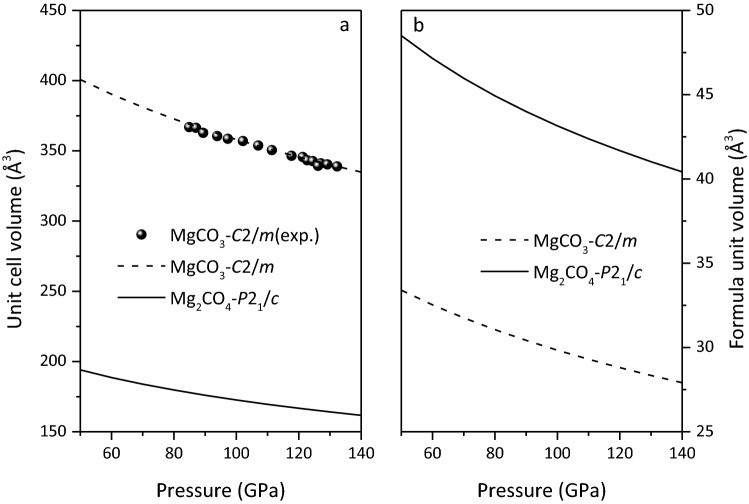
Equation of state for MgCO_3_-*C*2*/m* and Mg_2_CO_4_-*P*2_1_*/c*.

The equations of state for MgCO_3_-*C*2/*m* and Mg_2_CO_4_-*P*2_1_/*c* at 50 to 140 GPa are shown in Fig. 3. It is found that the equation of state of MgCO_3_-*C*2/*m* is in good agreement with available experimental data^6^. The equation of state of Mg_2_CO_4_-*P*2_1_/*c* is smaller than that of MgCO_3_-*C*2/*m*, and is almost parallel. The formula unit volume of MgCO_3_-*C*2/*m* is smaller than that of Mg_2_CO_4_-*P*2_1_/*c*, which is in line with their molecular formula composition relationship.

### Elastic properties

For monoclinic MgCO_3_-*C*2*/m* and Mg_2_CO_4_-*P*2_1_*/c*, there are 13 independent elastic constants ($c_{11}$, $c_{12}$, $c_{13}$, $c_{15}$, $c_{22}$, $c_{23}$, $c_{23}$, $c_{25}$, $c_{33}$, $c_{35}$, $c_{44}$, $c_{46}$, $c_{55}$ and $c_{66}$). The elastic constants are calculated using the stress–strain method^29^. In this work, all calculated elastic stiffness constants *c*_*ij*_ are checked using the mechanical stability criteria^34^ and find that they all meet the mechanical stability criteria in the studied pressure range, thus they are mechanically stable.

**Figure 4:**
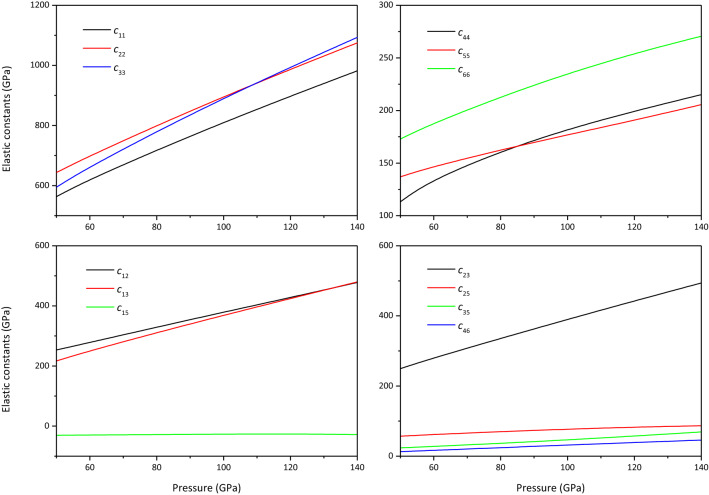
Elastic constants of MgCO_3_-*C*2*/m.*

**Figure 5:**
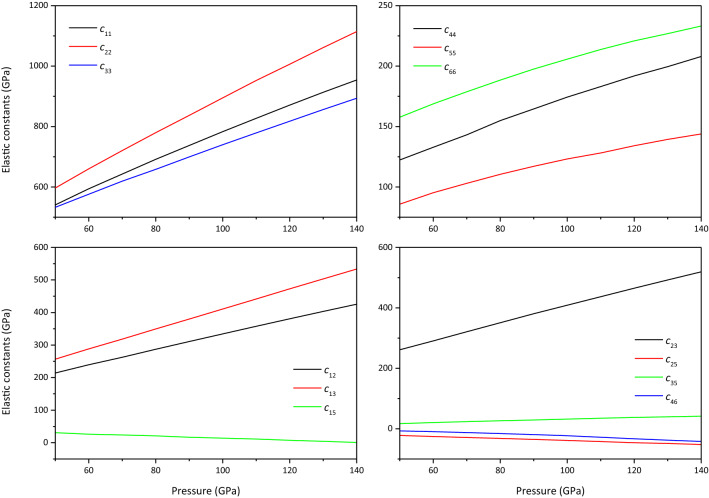
Elastic constants of Mg_2_CO_4_-*P*2_1_*/c.*

The elastic constants of MgCO_3_-*C*2*/m* and Mg_2_CO_4_-*P*2_1_*/c* are plotted in Figs. 4 and 5, respectively. It is found from Fig. 4 that at 50–110 GPa, $c_{22} > c_{33} > c_{11}$, indicating that the a-axis of MgCO_3_-*C*2*/m* is the most easily compressed, and the b-axis is the least compressed. At > 110 GPa, $c_{33} > c_{22} > c_{11}$, the c-axis is least likely to be compressed. From Fig. 5, it can be seen that $c_{22} > c_{11} > c_{33}$ in the studied pressure range, indicating that the c-axis of Mg_2_CO_4_-*P*2_1_*/c* is the most easily compressed, and the b-axis is the least compressible. In the previous study^13^, the elastic constants of MgCO_3_-$R\overline{3} c$ are consistent with the experimental results^21^. Therefore, the predicted elastic constants of MgCO_3_-*C*2*/m* and Mg_2_CO_4_-*P*2_1_*/c* in this work should be correct, but it needs to be further verified by experiments.

**Figure 6:**
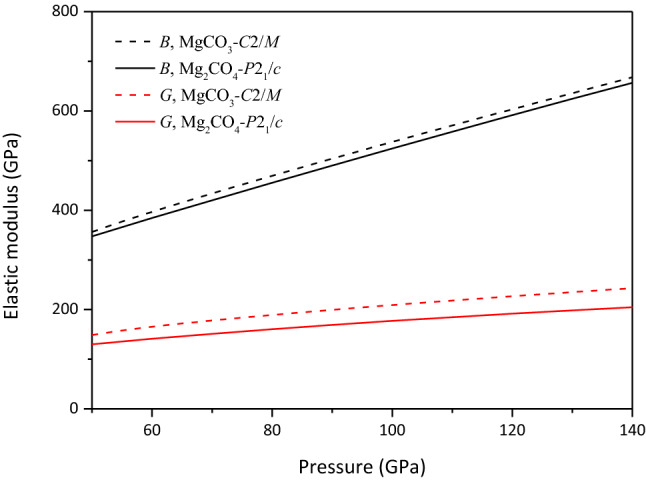
Bulk modulus *B* and shear modulus *G* of MgCO_3_-*C*2*/m* and Mg_2_CO_4_-*P*2_1_*/c*.

Based on the calculated elastic constants, the bulk modulus *B* and shear modulus *G* of MgCO_3_-*C*2*/m* and Mg_2_CO_4_-*P*2_1_*/c* are calculated using the Voigt-Reuss-Hill method^35–37^. As shown in Fig. 6, the bulk modulus *B* and shear modulus *G* of MgCO_3_-*C*2*/m* and Mg_2_CO_4_-*P*2_1_*/c* increase with increasing pressure, and the bulk modulus *B* and shear modulus *G* of MgCO_3_-*C*2*/m* are larger than those of Mg_2_CO_4_-*P*2_1_*/c* at 50–140 GPa.

### Seismic properties

Based on the calculated bulk and shear moduli and density, the compression (*V*_*P*_) and shear (*V*_*S*_) velocities of MgCO_3_-*C*2*/m* and Mg_2_CO_4_-*P*2_1_*/c* are given by the following expressions^38^:

1 $$V_{P} = \sqrt {\frac{3B + 4G}{{3\rho }}}$$

2 $$V_{S} = \sqrt {\frac{G}{\rho }}$$

**Figure 7:**
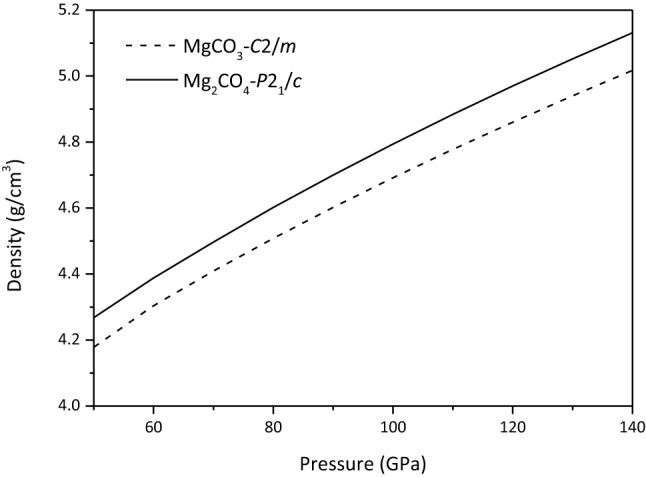
Density of MgCO_3_-*C*2*/m* and Mg_2_CO_4_-*P*2_1_*/c*.

**Figure 8:**
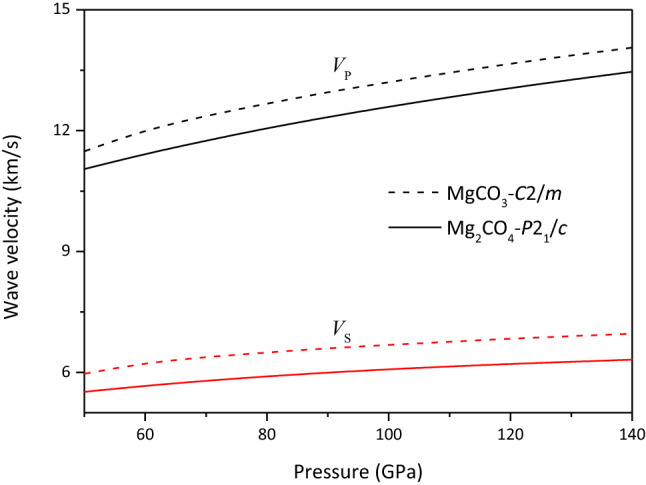
Compression (*V*_*P*_) and shear (*V*_*S*_) velocities of MgCO_3_-*C*2*/m* and Mg_2_CO_4_-*P*2_1_*/c*.

As shown in Fig. 7, the density of Mg_2_CO_4_-*P*2_1_*/c* is larger than that of MgCO_3_-*C*2*/m*, and the difference between their bulk modulus and shear modulus is smaller, respectively. Therefore, the *V*_*P*_ and *V*_*S*_ of MgCO_3_-*C*2*/m* are larger than those of Mg_2_CO_4_-*P*2_1_*/c* in the studied pressure range, and their *V*_*P*_ and *V*_*S*_ tend to be parallel, respectively (See Fig. 8).

**Figure 9:**
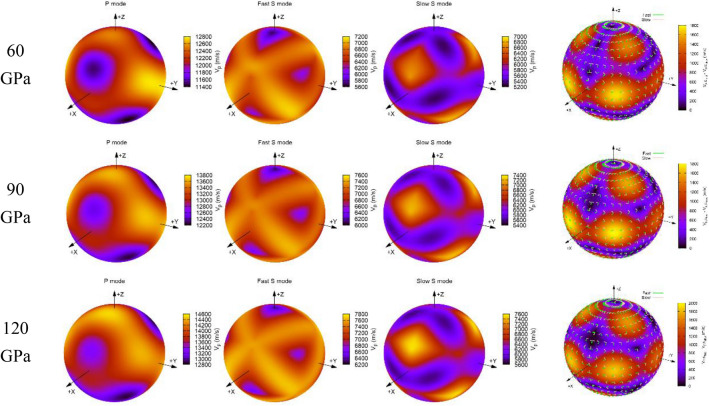
3D representation of the *V*_*P*_ and *V*_*S*_ and the shear wave splitting and polarization vectors of MgCO_3_-*C*2*/m* at various pressures.

**Figure 10:**
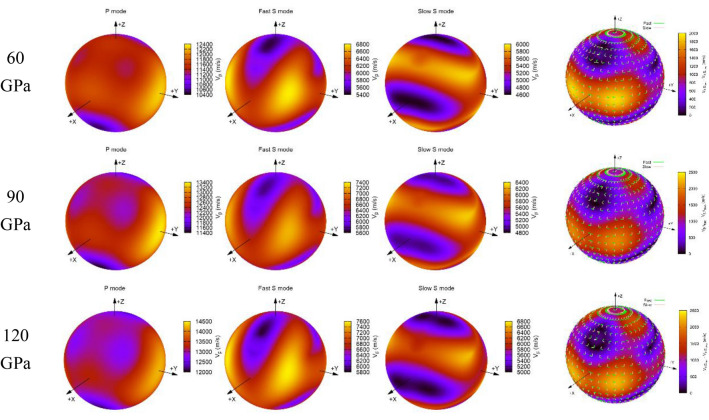
3D representation of the *V*_*P*_ and *V*_*S*_ and the shear wave splitting and polarization vectors of Mg_2_CO_4_-*P*2_1_*/c* at various pressures.

The *V*_*P*_ and *V*_*S*_ of MgCO_3_-*C*2*/m* and Mg_2_CO_4_-*P*2_1_*/c* along different directions can be obtained by solving the Christoffel equation^39^ $\left| {C_{ijkl} n_{j} n_{l} - \rho V^{2} \delta_{ik} } \right| = 0$. In order to visualize the propagation wave velocities of MgCO_3_-*C*2*/m* and Mg_2_CO_4_-*P*2_1_*/c* along different directions, the AWESoMe program^40,41^ is used to plot their 3D representations of *V*_*P*_ and *V*_*S*_ and shear wave splitting and polarization vectors at various pressures (Figs. 9 and 10).

The anisotropy *A*_*P*_ of the *V*_*P*_ for MgCO_3_-*C*2*/m* and Mg_2_CO_4_-*P*2_1_*/c* is defined as^42^:

3 $$A_{P} = \frac{{V_{P,\max } - V_{P,\min } }}{{V_{P,aggregate} }} \times 100\% .$$

The polarization anisotropy *A*_*S*_ of the *V*_*S*_ is defined as

4 $$A_{S} = \frac{{\left( {V_{S1} - V_{S2} } \right)_{\max } }}{{V_{S,aggregate} }} \times 100\% .$$

**Figure 11:**
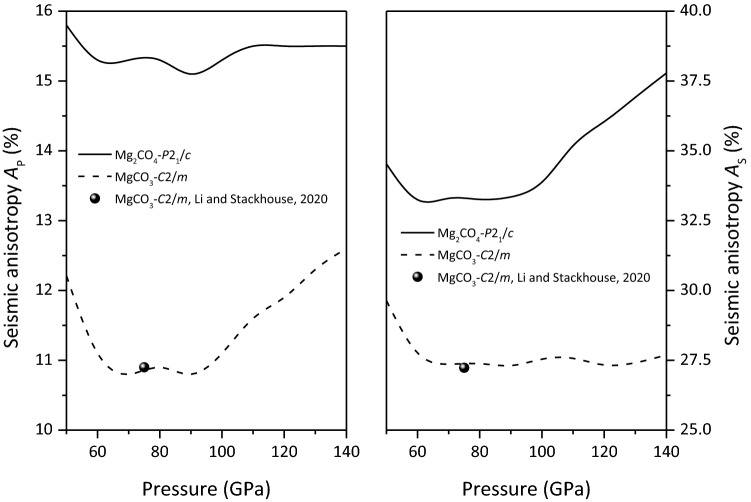
Seismic anisotropy of MgCO_3_-*C*2*/m* and Mg_2_CO_4_-*P*2_1_*/c*.

The seismic anisotropies of MgCO_3_-*C*2*/m* and Mg_2_CO_4_-*P*2_1_*/c* are shown in Fig. 11. The seismic anisotropy of MgCO_3_-*C*2*/m* at 75 GPa is found to be in good agreement with the previous theoretical calculations^10^. The anisotropy of Mg_2_CO_4_-*P*2_1_*/c* is obviously larger than that of MgCO_3_-*C*2*/m*. The seismic anisotropy of MgCO_3_-*C*2*/m* and Mg_2_CO_4_-*P*2_1_*/c* showed obvious nonlinear dependence on pressure. This is mainly due to the nonlinear pressure of wave velocity caused by the nonlinear pressure dependence of the elastic moduli of MgCO_3_-*C*2*/m* and Mg_2_CO_4_-*P*2_1_*/c*, especially their shear moduli.

Although the previous experimental^4–6^ and theoretical^7–13^ studies obtained MgCO_3_-*C*2*/m* at high temperature and pressure, they did not consider the reaction with MgO, the main mineral of the Earth's lower mantle. The theoretical^14^ and experimental^15^ results of Gavryushkin et al. show that MgCO_3_ reacts with MgO to form Mg_2_CO_4_-*P*2_1_/*c* orthocarbonate under the temperature and pressure conditions of the lower mantle. By comparing the high-pressure physical properties of MgCO_3_-*C*2*/m* and Mg_2_CO_4_-*P*2_1_/*c*, it is found that their seismic anisotropy is quite different, while the equation of state, elastic modulus, density and wave velocity have similar relationship with pressure. The low wave velocities of Mg_2_CO_4_-*P*2_1_/*c* are more suitable to explain the existence of low-velocity zone near the subducting slab. Therefore, we believe that Mg_2_CO_4_-*P*2_1_/*c* may exist in the deep mantle, providing strong evidence for carbon storage in carbonate minerals, which may be the main reason why carbonate rocks cannot be detected in the lower mantle.

### Minimum thermal conductivity

The thermal conductivity of minerals is critical to understanding the Earth's thermal balance and history^43^. The minimum thermal conductivity of MgCO_3_-*C*2*/m* and Mg_2_CO_4_-*P*2_1_*/c* are calculated using Cahill’s model:

5 $$K_{\min } = ({{k_{B} } \mathord{\left/ {\vphantom {{k_{B} } {2.48}}} \right. \kern-\nulldelimiterspace} {2.48}})n^{2/3} (v_{P} + 2v_{S} )$$

**Figure 12:**
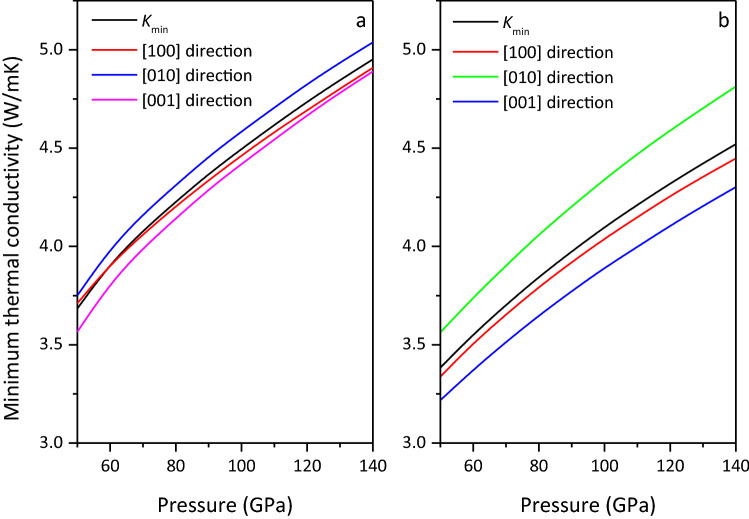
Minimum thermal conductivity of MgCO_3_-*C*2*/m* (**a**) and Mg_2_CO_4_-*P*2_1_*/c* (**b**).

The anisotropy of the minimum thermal conductivity can be calculated by changing Eq. (5) into the following form:

6 $$K_{min} = ({{k_{B} } \mathord{\left/ {\vphantom {{k_{B} } {2.48}}} \right. \kern-\nulldelimiterspace} {2.48}})n^{2/3} (v_{P} + v_{S1} + v_{S2} )$$

where *k*_*B*_ is Boltzmann’s constant, *n* is the atomic number density per unit volume. The minimum thermal conductivities of MgCO_3_-*C*2*/m* and Mg_2_CO_4_-*P*2_1_*/c* are shown in Fig. 12, and it is found that their minimum thermal conductivities increase with the increase of pressure, and that of MgCO_3_-*C*2*/m* is larger than that of Mg_2_CO_4_-*P*2_1_*/c*. In the studied pressure range, *K*_min_[010] > *K*_min_[100] > *K*_min_[001], indicating that the thermal conductivity in the [010] direction is the largest and the thermal conductivity in the [001] direction is the smallest.

### Thermodynamic properties

**Figure 13:**
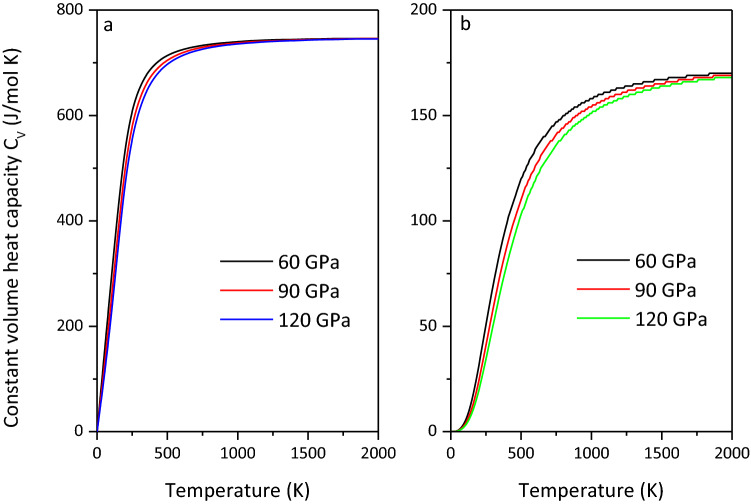
Constant volume heat capacity *C*_*V*_ of MgCO_3_-*C*2*/m* (**a**) and Mg_2_CO_4_-*P*2_1_*/c* (**b**).

**Figure 14:**
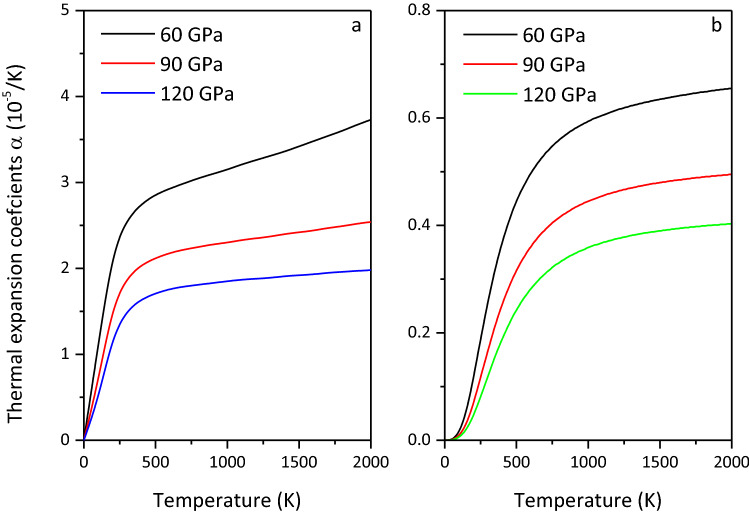
Thermal expansion coefficient $\alpha$ of MgCO_3_-*C*2*/m* (**a**) and Mg_2_CO_4_-*P*2_1_*/c* (**b**).

Thermodynamic parameters are the preconditions for deriving the thermal state of the Earth's interior. Therefore, the thermodynamic properties of MgCO_3_-*C*2*/m* and Mg_2_CO_4_-*P*2_1_*/c* are crucial for studying the thermal state of the lower mantle. The constant volume heat capacity (*C*_*V*_) and the thermal expansion coefficient ($\alpha$) of MgCO_3_-*C*2*/m* and Mg_2_CO_4_-*P*2_1_*/c* at various pressures are depicted in Figs. 13 and 14, respectively. The *C*_*V*_ and $\alpha$ of MgCO_3_-*C*2*/m* are larger than those of Mg_2_CO_4_-*P*2_1_*/c* under the same pressure.

## Conclusions

On the basis of verifying the structure and equation of state of MgCO_3_-*C*2/*m*, the phase transition pressure of Mg_2_CO_4_-*P*2_1_/*c* is determined. The high-pressure physical properties of MgCO_3_-*C*2/*m* and Mg_2_CO_4_-*P*2_1_/*c* at 50–140 GPa are investigated by first-principles calculations. By comparison, it is found that the elastic modulus and wave velocity of Mg_2_CO_4_-*P*2_1_/*c* are smaller than those of MgCO_3_-*C*2/*m*, and the density and seismic anisotropy are larger than those of MgCO_3_-*C*2/*m*. The low wave velocity of Mg_2_CO_4_-*P*2_1_/*c* may be more suitable to explain the existence of the low-velocity zone near the subducting slab. Therefore, it is believed that Mg_2_CO_4_-*P*2_1_/*c* may exist in the deep mantle, providing strong evidence for carbon storage in carbonates and the reason why it cannot be detected in the lower mantle. The minimum thermal conductivity of MgCO_3_-*C*2/*m* is greater than that of Mg_2_CO_4_-*P*2_1_/*c*, and their minimum thermal conductivity is the largest in the [010] direction and the smallest in the [001] direction. The constant volume heat capacity *C*_*V*_ and thermal expansion coefficient $\alpha$ of MgCO_3_-*C*2*/m* are larger than those of Mg_2_CO_4_-*P*2_1_*/c*. Unfortunately, there are no experimental data on the elastic constants, thermodynamic parameters, and minimum thermal conductivity of MgCO_3_-*C*2/*m* and Mg_2_CO_4_-*P*2_1_/*c*, so further verification is required.

## Data Availability

The datasets used and/or analysed during the current study available from the corresponding author on reasonable request.
